# Chagas disease affects the human placental barrier’s turnover dynamics during pregnancy

**DOI:** 10.1590/0074-02760210304

**Published:** 2022-06-27

**Authors:** Luciana Mezzano, Joana Paola Morán, María José Moreira-Espinoza, María Fernanda Triquell, Julieta Mezzano, Cintia María Díaz-Luján, Ricardo Emilio Fretes

**Affiliations:** 1 National University of Córdoba, Institute of Research in Health Sciences (CONICET), Medicine School, Cellular Biology Institute, Cathedra of Cellular Biology, Histology and Embryology, Córdoba, Argentina; 2 National University of Villa María, Institute of Human Sciences, Histology, Cytology and Embryology Department, Córdoba, Argentina; 3 Tufts University, Friedman School of Nutrition Science and Policy, Boston, MA, USA

**Keywords:** congenital Chagas disease, Trypanosoma cruzi, trophoblast turnover, Ki67 antigen, Syncytin-1 antigen

## Abstract

**BACKGROUND:**

*Trypanosoma cruzi* crosses the placental barrier and produces the congenital transmission of Chagas disease (CD). Structural alterations of the chorionic villi by this parasite have been described *in vitro*, but little is known about trophoblast turnover in placentas from women with CD.

**OBJECTIVE:**

To analyze the proliferation and fusion processes in placentas from women with CD.

**METHODS:**

Archived human term placenta paraffin-embedded blocks were used, from women with CD (CDP), and no pathology (NP). Immunohistochemistry tests were performed for Ki67 to calculate the proliferation index (PI) of cytotrophoblast (CTB) and Syncytin-1, a fusion marker of syncytiotrophoblast (STB). Hematoxylin/Eosin stained sections were employed to analyze STB percentages, STB detachment areas and syncytial knots quantity. Non parametric Student’s *t*-tests were performed (p < 0.05).

**RESULTS:**

Syncytial knots and STB detachment significantly increased in placental villi from the CDP group. STB percentage was significantly lower in the CDP group as well as the PI and Syncytin-1 expression significantly decreased in these placentas, compared with control (NP).

**CONCLUSION:**

Dynamic of trophoblast turnover is altered in placentas from women with CD. These changes may lead into a gap in the placental barrier possibly allowing the parasite entry into the chorionic villi.

The human placenta contributes to the fetal development during pregnancy playing essential functions such as acting as an immunological barrier, supplying nutrients, and regulating the hormonal and gas-waste exchange between the mother and the fetus.^1^ In order to protect the fetus from the attack of possible pathogens, the placental barrier needs to be maintained intact.^2^ It has been shown that the trophoblast plays a key role in the placental infection by *Trypanosoma cruzi*, the causal agent of Chagas disease (CD). This parasite can cross the placental barrier and be transmitted from mother to the fetus, causing the congenital transmission of the disease.^3^ The CD prevalence in South American pregnant women ranges from 4 to 52%, and only 0.1 to 5% of the women can transmit the infection to their fetuses.^4^ ^,^ ^5^ ^,^ ^6^

The maintenance of the structural and functional integrity of placental tissue involves a highly regulated cellular turnover, which depends on a delicate balance between the cell proliferation, differentiation and death.^7^ The placental villous trophoblast is composed of two cellular layers: the syncytiotrophoblast (STB) and the cytotrophoblast (CTB). The STB is the first placental layer that is in direct contact with the maternal blood and is maintained through the proliferation and fusion of underlying CTB.^8^ The input of cytotrophoblastic components is counterbalanced by a continuous release of apoptotic material from the STB to maternal blood, where STB nuclei are sequestered in clusters (syncytial knots) prior to extrusion, as part of a physiological epithelial turnover of the placenta.^9^ ^,^ ^10^ ^,^ ^11^ ^,^ ^12^

In this study, we analyzed the proliferation and fusion processes in villous trophoblast by using Ki67, one of the proliferation markers which is seen in the nucleus of proliferating cells,^13^ ^,^ ^14^ ^,^ ^15^ and Syncytin-1, a captive retroviral glycoprotein present in placental cells, which is involved in trophoblast cell fusion and syncytia formation, respectively.^8^ ^,^ ^16^ ^,^ ^17^ ^,^ ^18^

Some authors have demonstrated *in vitro* that *T. cruzi* induces cellular proliferation and differentiation in the trophoblastic cell line BeWo, and apoptosis in chorionic villi explants.^19^ ^,^ ^20^ ^,^ ^21^ On the other hand, previous work has also described that *in vitro T. cruzi* infection produces structural alterations in the chorionic villi, which in turns, reduces placenta defenses and may contribute to the vertical transmission of CD.^22^ Further studies have shown an increase in CTB proliferation index (PI) (by BrdU staining),^23^ as well as STB’s destruction and detachment in chorionic villi *in vitro* challenged with *T. cruzi*. This suggests that this is one of the mechanisms used by the parasite to infect and invade the human placenta and reach the fetal organism.^24^ However, little is known about proliferation and fusion events related with trophoblast renewal in placentas from women with CD. Therefore, the aim of this study was to analyze the proliferation and fusion processes involved in the human trophoblast turnover, in placentas from pregnant women with CD.

## SUBJECTS AND METHODS

*Placental samples* - Paraffin-embedded blocks of human term placentas (≥ 37 weeks) from histopathological archive were studied. These were obtained from Privado Hospital and University Hospital of Maternity and Neonatology in Córdoba, Argentina.

The normal placentas (NP; n = 9) used as a control group, came from full-term pregnant women with negative serology for Chagas or any other infection; without preexisting diseases or complications during pregnancy; whose deliveries were by cesarean section. The ages of the pregnant women were between 25 ± 6 years old. The weight and APGAR score of newborns were within normal limits.

The experimental group of placentas were obtained from full-term pregnant women with chronic Chagas disease (CCD)(CDP; n = 16) with negative serology for other infections, without pre-existing diseases. Deliveries were by cesarean section or normal labor at term.

*Histological technique* - The paraffin-embedded blocks of placental tissue were cut into 4 µm thick sections obtained every 30 µm at three depths,^25^ which were used for hematoxylin/eosin staining and immunohistochemistry. To perform the immunohistochemistry, sections were mounted on positively charged glasses (Biotraza). Sections were deparaffinized with xylene two times for 10 min and rehydrated with decreased graded series of ethanol (for 5 min each) and distilled water.

*Stereological analysis* - The morphometric analysis of syncytial area, STB detachment area and syncytial knots were performed in hematoxylin/eosin-stained sections. Digital images (TIFF format) were captured using Leica DM500 biological microscope (DM500, Leica, Wetzlar, Germany) at a final magnification of 400X. In each sample, 10 fields were processed in a systematic and random manner, using Fiji/ImageJ software.^26^ The analysis of the image obtained from the optical density (OD) was emitted in gray scale. Each level of gray was expressed by pixels that were converted to microns (µm^2^).

The percentages of STB area and STB detached area were calculated in relation to the total chorionic villi area of each microscopic field analyzed, with the following equation: STB area or STB detached area (µm^2^)/ Total chorionic villi area (µm^2^) of the field x 100.^24^

The syncytial knots were identified in the periphery of STB of the chorionic villi, as protruded structure with juxtaposed nuclei with chromatin highly condensed and dispersed or in the form of a dense peripheral ring.^27^ In each field, the number of syncytial knots present was manually quantified, in relation to the total chorionic villi area analyzed.

The morphometric analyses were performed by two different observers at different times, and the average score was used.

*Ki67 and Syncytin-1 Immunohistochemistry* - Deparaffinized and rehydrated placental tissue sections were subjected to antigen retrieval with sodium citrate buffer solution (pH 6.0) in a pressure cooker for 20 min, followed by inhibition of endogenous peroxidase with H_2_O_2_ (0.3%) for 10 min.

For the detection of Ki67 (RTU-Ki 67- MM1 NovocastraTM) as a CTB proliferation marker, a mouse monoclonal antibody against Ki67 and a Novolink Polymer Detection System (Leica Biosystems) was used. Unspecific antigens were blocked with 0.4% casein in phosphate-buffered saline (pH 7.4). The sections were incubated with Ki67 primary antibody at a dilution of 1:400, overnight, in a wet chamber. Subsequently the sections were incubated with anti-mouse rabbit IgG (Post Primary) for 30 min and finally with Poly-HRP-IgG anti-rabbit (Polymer) for 30 min at 37ºC.

For the detection of Syncytin-1 (Z-25: sc-13088, Santa Cruz Biotechnology, INC.) a purified rabbit polyclonal antibody and a VECTASTAIN Elite ABC System Peroxidase, Rabbit IgG (Vector Laboratories) were used. The protein detected by this antibody corresponds to the ERVWE1 gene, an envelope protein that is mainly expressed in STB and is involved in the fusion of the CTB cells to form the STB. It is predicted to undergo post translational cleavage into a surface subunit and a transmembrane subunit. Sections were incubated with normal goat serum for 20 min, then with the Syncytin-1 primary antibody, dilution 1:200 overnight at 4ºC, and finally 30 min with the biotinylated secondary antibody anti-rabbit, followed by incubation for 30 min with Avidin-HRP at 37ºC in wet chamber.

Sections were revealed with DAB chromogen 3,3-diaminobenzidine (DAB Substrate Kit, Peroxidase (HRP) (Vector Laboratories) for three min to develop peroxidase activity, and then were counterstained with Hematoxylin (Biopur) for 20 s. Slides were mounted with DPX (Sigma-Aldrich). Negative controls were performed omitting the primary antibody.

To quantify Ki67 and Syncytin-1 expressions, 10 digital images were taken at random from each slice of placental tissue, obtained by a light video microscope (DM500, Leica, Wetzlar, Germany) at 400X. Images were processed in TIFF format and measurement was performed using computer assisted analysis Fiji/Image J software.^26^

Ki67 expression was evaluated by manually counting positive CTB cells in each analyzed image, and expressed as a PI, that was calculated considering the number of Ki67 positive marked CTB nuclei in 10.000 μm^2^ of analyzed chorionic villi area.^28^ CTB cells were identified by their location below the STB, of which nuclei was considered as a spherical or oval structure with partial or total immunostaining, with or without nucleolus present located at the periphery of the placental villi, adjacent to or fused with the STB layer.^7^

Syncytin-1 protein expression was evaluated in immunostained images using an ImageJ Fiji software color deconvolution plugin (version 1.2), that digitally separated DAB and hematoxylin staining and calculated their contribution, based on stain-specific redgreen-blue (RGB) absorption.^29^ The analysis of the image obtained from the OD was emitted in gray scale. Each level of gray was expressed by pixels that were converted to microns (µm^2^). This turned the brown color of the expression of Syncitin-1 into gray, in a scale that extends from 0 to 255 (0 corresponding to white and 255 to black). The values obtained were recorded as the average staining intensities in gray scale units for all threshold µm^2^. The expression was quantified by calculating the percentage (%) of positive Syncytin-1 immunostaining area (µm^2^)/total chorionic villi area analyzed (µm^2^) and expressed as % area/µm^2^.

*Statistical analysis* - Statistical analyses were performed using GraphPad Prism software version 7 (GraphPad Software, CA). Non parametric Student *t* test and post hoc “Mann Whitney test” was used to assess the effects of the CDP versus NP. Data are presented as means ± SEM, p values of < 0.05 were considered significant.

*Ethical statement* - This work was approved by the Health Research Ethics Assessment Council (COEIS) and the Health Research Ethics Institutional Committees (CIEIS) of the National Hospital of Clinic of the National University of Córdoba (UNC), No. 2710. Procedures are in agreement with the Helsinki Declaration of 1975 and revised in 1993.

## RESULTS

*Trophoblast analysis* - Analyses were performed to the terminal placental villi, characterized by their high degree of capillarization representing the main site of maternal-fetal exchange in images obtained from H/E stained placental sections. The syncytial knots quantity, STB area and STB detachment area percentages were measured in relation to the total chorionic villi are analyzed, to detect structural alterations in the placental trophoblast.

A significant increase of 64.5% (p = 0.0003) in syncytial knots number in CDP villi (0.076 ± 0.008, n = 12) was observed compared with normal placentas (0.027 ± 0.004, n = 7).

**Fig. 1:**
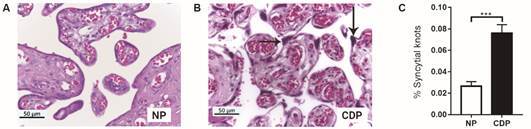
syncytial knots in Hematoxylin/Eosin stained placental villi sections. (A) Normal human placental villi (NP) and (B) term placental villi from women with Chagas disease (CDP) (Original magnifications: 400X; Arrows: syncytial knots); (C) percentage of syncytial knots which were analyzed with respect to total chorionic villi analyzed area. Image quantification was performed by Fiji ImageJ software; values shown are the mean ± SEM (CDP: n = 12, NP: n = 7, Student’s *t*-test and post hoc Mann Whitney *test*; ***p < 0.001).

This significant increment in syncytial knots quantity in CDP placental villi, could represent a rise in apoptosis processes in placentas from women with CD (Fig. 1A-C).

**Fig. 2:**
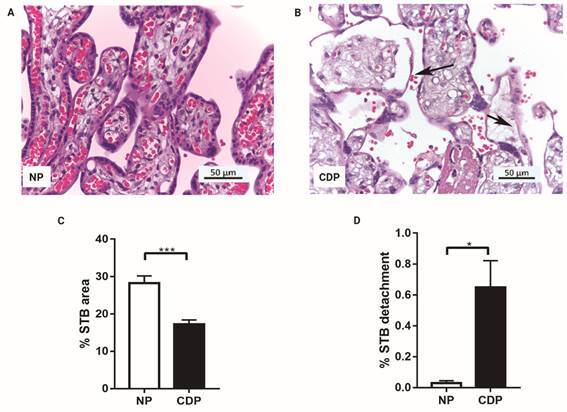
syncytiotrophoblast (STB) area and STB detachment area in Hematoxylin/Eosin stained placental villi sections. (A) Normal human placental villi (NP) and (B) term placental villi from women with Chagas disease (CDP) (Magnifications: 400X; arrows: STB detachment), (C) percentages of STB area (CDP: n = 16, NP: n = 4), and (D) STB detachment area (CDP: n = 12, NP: n = 7), calculated with respect to the total chorionic villi analyzed area (µm^2^). Image quantification was performed by Fiji ImageJ software; values shown are the mean ± SEM (Student’s *t*-test and post hoc Mann Whitney *test*; *p < 0.05).

With respect to the percentage of STB area, it was observed a significant decrease of 39% (p = 0.0004) in STB area in CDP placentas (17.2 ± 1.18, n = 16), compared with normal ones (28.2 ± 1.94, n = 4) (Fig. 2A-C).

STB detachment area percentage was studied. A significant increase of 95% (p = 0.0147) in STB detachment area in CDP placental villi (0.649 ± 0.172, n = 12) versus NP (0.0293 ± 0.0149, n = 7) was observed (Fig. 2A-D). This increment in the detached areas could be related with the decrease in STB area that was observed in CDP chorionic villi.

**Fig. 3:**
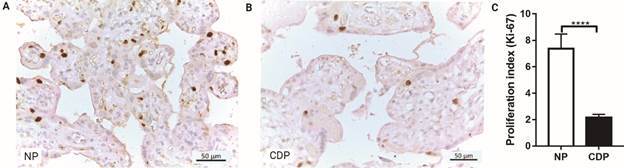
immunohistochemistry for Ki-67 monoclonal antibody in paraffinembedded placental villi sections. (A) Normal human placental villi (NP) and (B) term placental villi from women with Chagas disease (CDP) (Magnification: 400 X); (C) proliferation index: Ki-67 positive marked CTB nuclei/10.000 µm^2^ of analyzed area. Image quantification was performed by Fiji ImageJ software, values shown are the mean ± SEM (CDP: n = 14; NP: n = 8, Student’s *t*-test and post hoc Mann Whitney *test*; ****p < 0.0001).

*PI (Ki67 expression)* - A PI was calculated considering the number of CTB nuclei which were positive for Ki67, in 10.000 μm2 of the analyzed area. We observed that the PI was significantly lower (p < 0.0001) in CDP placental villi (2.16 ± 0.23, n = 14) than in NP (7.37 ± 1.11, n = 8), registering a decrease in Ki67 expression of 70.7% in CDP placental villi compared to normal controls (Fig. 3A-C).

**Fig. 4:**
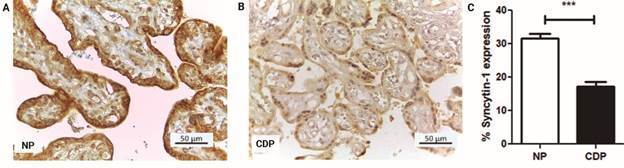
immunohistochemistry for Syncytin-1 monoclonal antibody in paraffinembedded placental villi sections. (A) Normal human placental villi (NP) and (B) term placental villi from women with Chagas disease (CDP) (Magnification: 400 X), (C) Syncytin-1 expression was measured as percentage (%) of positively marked area for Syncytin-1, with respect to total chorionic villi analyzed area (µm^2^). Image quantification was made by Fiji ImageJ software, values shown are the mean ± SEM (CDP: n = 14, NP: n = 9, Student’s *t*-test and post hoc Mann Whitney *test*; ****p < 0.0001).

*Trophoblast fusion process (Syncytin-1 expression)* - Syncytin-1 expression was located in the same areas either in CDP and NP placentas, but there was a significant decrease (p ≤ 0.0001) in their immunostaining in the trophoblast of CDP placentas of about 45.25% (17.3 ± 1.35, n = 14) compared to NP (31.6 ± 1.42, n = 9) (Fig. 4A-C).

## DISCUSSION

Previous studies have shown the important role played by the placental barrier´s first structure and the trophoblast´s turnover in the infection of the placenta by *T. cruzi* to limit the congenital transmission by this route. This can partly explain the low occurrence of vertical transmission of CD.^22^

*Trypanosoma cruzi* induces damage in the placental tissue. The most evident is the trophoblast detachment seen *in vitro* studies that leaves areas of denuded placental villi which would give the parasite a chance to infect the fetus.^2^ ^,^ ^30^ ^,^ ^31^ In placentas from women with CD we observed the same structural alterations with a significant increase of STB detachment and syncytial knots that could represent an apoptosis increment in chagasic placental villi. The increased areas of detached STB were also demonstrated in pregnancies complicated with pathologies such as preeclampsia, diabetes and fetal growth restriction, altogether with increased trophoblast apoptosis.^32^ According to the results presented in this work, it seems probable that the increase in the detachment of the STB chorionic villi and the decrease in STB areas could increase the possibility of tissue infection by *T. cruzi.*

Altemani et al.^33^ have observed severe histopathological changes, such as extensive necrosis, inflammatory infiltrate, and amastigote nests in placentas from mothers with acute CD with high parasitemia.^33^ Contrarily, these alterations were not described in placentas from mothers with CCD, in which STB detachment was also observed, in concordance with our results.^34^

*In vitro* studies have analyzed the interaction of *T. cruzi* with normal human chorionic villi explants that could be considered as the first interaction between the placenta and the parasite, in a short period of time.^30^ On the contrary, in this work we observed the effect of chronic Chagas on placentas from women who have suffered from this disease in the course of their pregnancy.

Some of our results are in accordance, and others differ, with those from *in vitro* studies. We show that, in placentas from women with CD, the turnover of trophoblast was modified through increasing STB detachment and formation of syncytial knots, alterations which have also been described in *in vitro* studies.^22^ ^,^ ^24^ ^,^ ^31^ ^,^ ^35^ As a consequence, it seems that changes in the turnover of the trophoblast start from the beginning of the infection (*in vitro* studies) and are reinforced during the course of the pregnancy (results of this work). However, little is known about the participation of the parasitemia and the immunologic system, genetic constitution of the mother and placenta, lineage of the *T. cruzi* and different possible answers of the placenta against infection. Furthermore, it would be necessary to corroborate results obtained in other *in vitro* models in placentas from women with CD, such as chorionic villi apoptosis, which was not the objective of this work.^20^ ^,^ ^35^ ^,^ ^36^

In previous works it was studied that *T. cruzi* produces structural^37^ ^,^ ^38^ and physiological changes of the placental barrier through nitric oxide synthase and nitrosative oxidative stress, which depend on the amount of parasites in the intervillous space.^24^ ^,^ ^39^ These alterations and the ones observed in the present work might allow the parasite to invade susceptible cells of the chorionic villi**.** However, it was also demonstrated that there was a clearance of parasites in the intervillous space and in the chorionic villi due to nitric oxide and nitrosative-oxidative stress, depending on the strain of the parasite.^22^ ^,^ ^24^ The surviving capacity of the *T. cruzi*´s cell and the full capacity of the trophoblast´s renewal, might condition the infection of the chorionic villi.

The proliferation of CTB is a critical event for the maintenance of the trophoblast. The normal epithelial turnover of the trophoblast is one mechanism that assures the integrity of this anatomical barrier. In the present study we evaluated the CTB proliferation by immunohistochemistry for Ki67 antibody in placental villi sections, where we observed a significant decrease in CTB PI (Ki67 expression) in placentas from women with CD. In contrast, in *in-vitro* models, other authors have observed that *T. cruzi* infection induces cellular proliferation in BeWo cells,^21^ and in placental trophoblast explants, suggesting that the placental turnover could be considered to be a defense mechanism against pathogens.^23^ In this work, this process is investigated for the first time in placentas from pregnant women with CD, showing that it could be different from what is observed *in vitro*.

Fusion of CTB into the multinucleated STB layer is an essential step for the development of a functional placenta.^10^ The gene of the human endogenous retrovirus Syncytin-1, is a key factor for mediating cell-cell fusion of CTBs.^40^ ^,^ ^41^ Clinically, it has been studied that a decrease in the normal level of mRNA for Syncytin-1 is associated with placental dysfunction.^16^ It was described that low Syncytin-1 expression, in both cultured CTB and primary tissues from pathological placentas, supports an intrinsic placenta-specific deregulation of cell-cell fusion in the formation of STB leading to increased apoptosis.^42^ In the present study, the Syncytin-1 expression was analyzed by immunohistochemistry as a fusion marker in placental villous sections. A significant decrease in the fusion rate was observed in the placentas of women with CD.

The decrease in Syncytin-1 expression observed in infected placentas has also been described by other authors in studies performed in intrauterine growth restriction and in preeclamptic placentas, where a decreased expression of Syncytin-1 mRNA and protein and in fusion index was also observed.^8^ ^,^ ^42^ The regulatory mechanisms of these processes should be studied to detect any modulatory factor that explains this relationship, since it is known that Syncytin-1 has the ability to block their own receptor to modulate its activity.

The presence of Syncytin-1 in syncytial knots could be related to modulation and activation of immune cells.^43^ ^,^ ^44^ It was seen that Syncytin-1 plays a role in the suppression of the maternal immune system,^45^ therefore the decrease in its expression in CDP could be associated with inflammatory processes observed in some placentas of women with Chagas.^33^ The placenta is a rich source of exosomes which are related to fetal allograft survival, and resistance to specific infections, among other functions.^46^ ^,^ ^47^ Tolosa suggests that the presence of Syncytin-1 in placental exosomes might provide a mechanism for this protein to reach and interact with target cells of the maternal immune system during pregnancy.^45^ It is highly probable that placenta-derived exosomes play essential roles also during *T. cruzi* interplay since the parasites increase exosomes in chorionic villous explants,^48^ topics that would be very interesting to explore.

Syncytin-1 maintains the balance between the CTB pool and the STB layer during placental development.^49^ If Syncytin-1 protein is insufficient, cell cycle arrest may occur in CTBs. According to our results, the decrease in the CTB PI and the absence of continuous fusion with STB may result in impairment of the STB layer structure and function. It was also observed that decreased levels of Syncytin-1 protein were accompanied by an increase in apoptosis, as was seen in BeWo cells as well as in preeclamptic human placentas.^50^ Thus, changes in cell cycle progression and apoptosis caused by altered Syncytin-1 expression may cause abnormalities in CDP as it was seen in preeclampsia cases.

The alterations in the dynamics of trophoblast renewal observed in this work might have a direct repercussion on the clinic history of newborns by affecting its intrauterine development, or even the development of pathologies in its adult life.^51^ However, this clinical association was not the objective of this work, being an interesting topic that would require a deeper analysis in future works.

*Trypanosoma cruzi* alters the normal morphology of the placental structure, both in renewal processes and also causing damage on trophoblast chorionic villi. Any process disturbing the balance between proliferation and syncytial fusion will alter the integrity and function of the layer as a whole, which in turn may impair gas and/or nutrient transfer to the fetus as well as endocrine activities.^52^ This study provides new data on changes in human trophoblast turnover caused by *T. cruzi in vivo*, affecting proliferation, fusion, and STB renewal. In the present study we observed a significant decrease in the proliferation of CTB and the fusion of STB in placentas from mothers with CD compared with normal placentas from clinically healthy women. This may tell us that there is a placental deficiency to repair the damage induced by *the* parasite that could be of crucial importance for the study of congenital transmission. Our results, in agreement with previous studies, suggest that the renovation of the trophoblast would be altered in placentas from women with CD, and these alterations could be due to changes in the dynamics of the trophoblast renewal. These changes may lead into a gap in the placental barrier allowing the parasite’s entry into the fetal blood. Based on these results we conclude that *T. cruzi* infection modifies the human trophoblast turnover in placentas from pregnant women with CD, altering their first processes, as the proliferation and CTB fusion, and the last steps, as the STB apoptosis (syncytial knots) and STB detachment.
